# Substance Addiction Rehabilitation Drugs

**DOI:** 10.3390/ph17050615

**Published:** 2024-05-10

**Authors:** Shu Yuan, Si-Cong Jiang, Zhong-Wei Zhang, Zi-Lin Li, Jing Hu

**Affiliations:** 1 College of Resources, Sichuan Agricultural University, Chengdu 611130, China; zzwzhang@126.com; 2 Haisco Pharmaceutical Group Comp. Ltd., Chengdu 611138, China; bloodyshadow@foxmail.com; 3 Department of Cardiovascular Surgery, Xijing Hospital, Medical University of the Air Force, Xi’an 710032, China; lizhuoyuanbb@163.com; 4 School of Medicine, Northwest University, Xi’an 710069, China; hujinglzbx@163.com

**Keywords:** dopaminergic neurons, GABAergic neurons, serotoninergic neurons, substance addiction, synaptic plasticity

## Abstract

The relapse rate of substance abusers is high, and addiction rehabilitation adjunct drugs need to be developed urgently. There have been numerous reports on blocking the formation of substance addiction, but studies on drugs that can alleviate withdrawal symptoms are very limited. Both the dopamine transporter (DAT) hypothesis and D3 dopamine receptor (D3R) hypothesis are proposed. DAT activators reduce the extracellular dopamine level, and D3R antagonists reduce the neuron’s sensitivity to dopamine, both of which may exacerbate the withdrawal symptoms subsequently. The D3R partial agonist SK608 has biased signaling properties via the G-protein-dependent pathway but did not induce D3R desensitization and, thus, may be a promising drug for the withdrawal symptoms. Drugs for serotoninergic neurons or GABAergic neurons and anti-inflammatory drugs may have auxiliary effects to addiction treatments. Drugs that promote structural synaptic plasticity are also discussed.

## 1. Introduction

The global number of substance abusers has reached 296 million [1]. Despite the benefits that prescription substances(mainly opioids) offer in acute pain management, the abuse and overuse of these agents have contributed to significant health and economic burdens for the patients, their families, and society [2,3]. The comorbidity of mental disorders and substance abuse has now been recognized universally. And the prevention of the development of secondary conditions as a consequence of primary disorders should reduce the impact of these conditions on both the individual and society [4,5].

However, numerous basic medical studies and clinical observations have shown that substance addiction is not only a bad habit, but also a recurrently chronic brain disease. Substance addicts often have very high relapse rates. The abstinence rates from cocaine for the patients treated with aversion therapy were 68.6% and 53% at six months and one year after the treatments, respectively [6]. A survival analysis of substance abuse relapse found that, in the first six months, the cumulative survival rate was 83%; however, after 24 months, it was 46% and the following time was consistent [7]. Once stopped, there will be an abstinence syndrome (withdrawal symptoms) such as anxiety, sudden changes in temperature, tears, runny nose, sweating, nausea, vomiting, abdominal pain, and diarrhea [8,9,10,11]. Repeated substance abuse induces neuroplastic alternations in dopaminergic neurons in the striatum and midbrain, increasing the neuron’s response to the substance rewards, decreasing the susceptivity to non-substance cues and enhancing the susceptivity to tense and dysphoric stimuli [12]. The functionally and morphologically related brain structures receiving and interpreting stimuli correlated with positive feeling, satisfaction, and addiction are defined as the brain reward system. Addictive substances are strong rewarding stimuli. Once the reward is delayed, the abuser will have a psychological desire to take the substance, resulting in the relapse behavior [13,14,15,16]. Therefore, interrupting the reward pathway and alleviating withdrawal symptoms are the keys to successful substance abuse rehabilitation.

## 2. Dopamine Transporter Hypothesis

**Figure 1:**
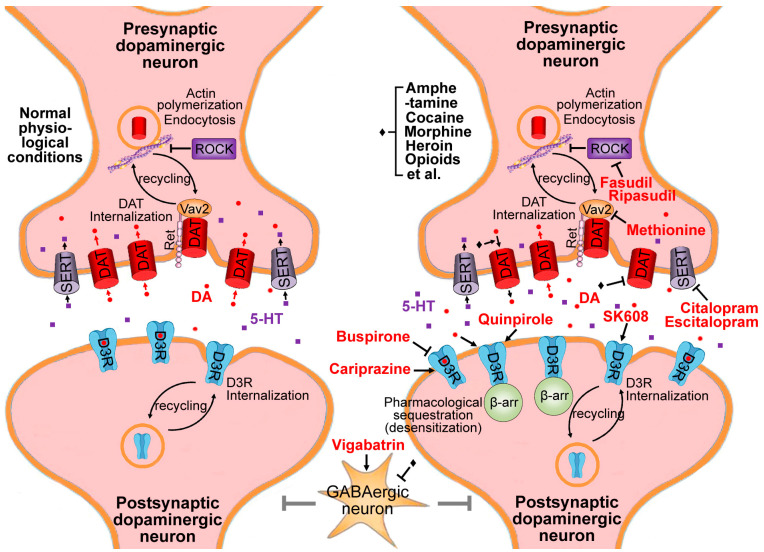
Substance-induced extracellular dopamine accumulation and putative treatment drugs. Dopamine (DA), dopamine transporter (DAT) activators, D_3_ dopamine receptor (D3R) antagonists and agonists, serotonin transporter (SERT) inhibitors, and GABA transaminase inhibitors are marked with the red color. Cocaine inhibits DAT directly. Amphetamine reverses the direction of dopamine transport. Rho-family guanine nucleotide exchange factor protein Vav2 was required for DAT cell surface expression. L-methionine down-regulates *Vav2* gene. Rho-associated protein kinase 1 (ROCK) inhibitors (fasudil and ripasudil) may promote DAT recycling by enhancing cell surface expression in an actin polymerization-dependent manner. D3R is dramatically up-regulated following chronic substance abuse, while D3R antagonists (e.g., buspirone) produce a blockade of substance-primed reinstatement. Both dopamine and D3R partial agonists significantly induce D3R pharmacological sequestration and desensitization, where β-arrestin is involved. Partial D3R agonists would compensate the low dopamine tone and reduce craving during the withdrawal. The D3R partial agonist SK608 down-regulates D3R protein steady-state level but does not induce pharmacological sequestration, and, therefore, may be an ideal drug for addiction rehabilitation. Cocaine also increases 5-hydroxytryptamine (5-HT; serotonin) efflux. Serotonin reuptake inhibitors citalopram and escitalopram reversed the compulsion to cocaine. Opioids elicit robust dopamine transients through the derepression of dopamine neurons via inhibiting GABAergic neurons. Vigabatrin, an irreversible GABA transaminase inhibitor, attenuates the release of dopamine upon substance challenges.

Dopaminergic neurons in the nucleus accumbens (NAc) and the ventral tegmental area (VTA) play an important role in the perception of rewarding stimuli, such as the rewards related with substance addiction [13,17]. After cocaine treatments, the extracellular dopamine (DA) contents increased dramatically. DA reuptake regulated by dopamine transporters (DATs) is a crucial mechanism for physiological DA homoeostasis (Figure 1). Cocaine inhibits DAT directly, while amphetamine is a substrate of DAT and reverses the direction of dopamine transport [18]. Other than cocaine-like stimulants, amphetamine stimulates the endocytosis of DAT in the plasmalemma (DAT internalization). The decreased content of DAT on the plasmalemma, in turn, promotes extracellular dopamine accumulation [19,20], which might be another reason that causes addiction to amphetamine (Figure 1).

A study showed that the Rho/Rac GTPase activator Vav2 plays an irreplaceable role in DAT expression in the plasmalemma and co-regulates the DAT activity by binding with a glial-cell-line-derived neurotrophic factor (GDNF) receptor Ret (Figure 1). Vav2 or Ret knocked-out mice showed enhanced DAT activities, accompanied with an enhancement in intracellular DA levels in NAc. These knocked-out mice treated with cocaine displayed diminished behavioral cocaine responses [21]. But all known DAT modulators [22] or Vav2 antagonists are non-FDA-approved drugs. Interestingly, recent evidence highlighted that piRNAs induced by L-methionine down-regulate the Vav2 gene via a miRNA-like mechanism (Figure 1). Accordingly, methionine has been proven to show therapeutic effects for cocaine addiction [23,24]. L-methionine blocked the sensitization to the locomotor-activating effects of cocaine and attenuated the substance-primed reinstatement also by influencing DNA methylation in NAc [25].

On the other hand, dynamins control endocytic DAT recycling in an interaction with actins in the cytoskeleton [26]. The Rho-associated protein kinase 1 (ROCK) was significantly induced in the NAc of animal models of addiction. The micro-injection of ROCK inhibitors blocked the habitual responses to cocaine in the actin polymerization-mediated process [27], suggesting that blocking ROCK may promote DAT recycling (enhancing cell surface expression) [28] and change the context of cocaine habits (Figure 1). Thus far, only two FDA-approved ROCK inhibitors, fasudil and its derivative ripasudil, showed therapeutic effects to some neurological diseases [29,30]. Fasudil improved spatial learning and memory capacity and decreased the burst of smoking-induced inflammatory factors in the hippocampus [31].

However, most of the above studies have focused on the formation mechanism of substance addiction, without involving the rehabilitation treatments of long-term substance abusers. The fact that the brain has adapted to high extracellular dopamine levels can subject the patient to frequent substance self-administration to maintain a normal sensation (the level of dopamine secreted under normal physiological conditions is no longer sufficient to maintain a normal neural activity). At this point, strengthening dopamine reuptake (activating DAT) or reducing the extracellular dopamine level may exacerbate withdrawal symptoms subsequently. Therefore, these drugs may not be adopted during the substance abuse rehabilitation.

## 3. D_3_ Dopamine Receptor Hypothesis

Recently, D_3_ dopamine receptors (D3Rs) have attracted much attention as targets for developing medicines to treat substance addiction. D3Rs plays an essential role in cocaine addiction because they are selectively distributed in neural circuits where the rewarding pathways are, and, also, because D3R is involved in the behavioral responses to the substance [32]. D3R-regulated plasticity in the ventral pallidum drives extracellular dopamine accumulation in the NAc during relapse to cocaine after the withdrawal [33]. Different from D2Rs, D3Rs are dramatically increased after long-term substance abuse. A post-mortem human study has shown that D3R was enhanced in the NAc of patients with a cocaine overdose [34]. Positron emission tomography (PET) studies have indicated an enhancement in the D3R expression in the substantia nigra, hypothalamus, and amygdala of the patients with a history of cocaine [35]. And, in a rhesus monkey model, PET imaging confirmed that the utility of the D3R sensitivity, but not the D2R availability, is a biochemical marker for vulnerability and resilience to cocaine [36]. It has been suggested that D3R increasing may be a consistently predictive marker and a clinically therapeutic target for addiction [37].

### 3.1. D3R Antagonists

Accordingly, D3R antagonists may reduce substance dependence and relapse behaviors [37,38,39]. For instance, the micro-injection of D3R antagonist SB-277011 into the NAc and the central amygdala repressed contextual-cue-derived cocaine seeking in rats [40]. The D3R antagonist PG01037 attenuated opioid-induced hyper-locomotion and anti-nociception in mice [41]. Among D3R antagonists, buspirone has been originally characterized as a selective serotonin 1A (5HT1A) receptor partial agonist and approved as an anxiolytic for over 25 years [42]. Besides 5HT1A receptors, both D3R and D4R may also be occupied by buspirone at pharmacologically relevant doses [42,43]. Buspirone produced a dose-dependent, apparent blockade of methamphetamine-primed and cue-induced reinstatement (Figure 1) [42]. In a cocaine self-administration study with rhesus monkeys, buspirone significantly abolished responding for cocaine at doses that did not affect the response to foods consistently [44]. Buspirone significantly reduced alcohol intake in the two-bottle choice paradigm [45]. However, currently, most D3R antagonists show a low physical property, poor bioavailability, or metabolic instability, and, therefore, may not be used as clinical drugs for addiction [37]. Furthermore, some D3R antagonists exhibited unacceptable increases in blood pressure in the presence of cocaine, because kidneys have high levels of D3Rs [46]. On the other hand, D3R antagonists may reduce the neuron’s sensitivity to dopamine and may exacerbate withdrawal symptoms subsequently. Therefore, D3R antagonists may not be suitable for substance abuse rehabilitation. For example, a multisite, randomized, double-blind clinical trial indicated that buspirone was unlikely to have beneficial effects on preventing a relapse to cocaine and may worsen the outcomes in cocaine-dependent women [47].

There exists a contradiction: since the density of dopamine receptors on the postsynaptic membrane of substance abusers are increased, they should be more sensitive to dopamine changes and, therefore, are less likely to experience withdrawal symptoms. But the fact is exactly the opposite: long-term abusers develop a greater physical and psychological dependence on the substance.

The internalization (endocytosis) of G-protein-coupled receptors has been suggested to be an initial step in receptor lysosomal degradation and endocytic receptor recycling. Nevertheless, the D3R internalization is an exception where, upon dopamine treatment, D3R becomes desensitized, and this process is thus called pharmacological sequestration. This process has been described as the sequestration of membrane receptors into more hydrophobic fractions within the plasmalemma without being subjected into endocytosis. Pharmacological sequestration would render the receptors in a desensitized state inside of the plasmalemma [48,49]. β-arrestin is involved in this desensitization process (Figure 1) [50,51,52]. The 1 μM dopamine treatment at 37 °C for 1 h resulted in a 20% increase in the D3R protein steady-state level, but the same treatment led to 30% D3R proteins undergoing pharmacological sequestration, while 10 μM dopamine caused 40% desensitization [49]. Taken together, the excessive accumulation of extracellular dopamine may lead to a decline in effective D3R proteins (Figure 1). Pharmacological sequestration prevents D3R from entering the lysosomal pathway [53], which may be another important reason for the increase in D3R caused by long-term substance abuse.

### 3.2. D3R Partial Agonists

The fact that dopamine reduces effective D3R proteins leads to another interesting phenomenon: in addition to D3R antagonists, its agonists can also treat substance addiction. D3R partial agonists showed therapeutic effects to cocaine in non-human primates and rodents of relapse-like substance-seeking behavior [37,54,55,56,57,58,59]. Although a clinical trial showed that the dopamine D2/D3 receptor agonist quinpirole (an FDA-approved D3R agonist) had no significant effect on the place preference induced either by cocaine or morphine [60], cariprazine (another FDA-approved D3R agonist) significantly decreased cocaine self-administration in rats [61]. Studies have proven that, after substance abuse, when the extracellular dopamine level is enhanced, partial agonists competed with the endogenous agonists to bind with the receptors, relieving the symptoms correlated with substance abuse. Nevertheless, during withdrawal, when the extracellular dopamine level has largely declined, partial agonists compensated the low dopamine tone, maintaining normal dopaminergic neuron function and reducing substance-seeking behaviors [37]. Moreover, partial agonists usually have lesser addiction liability than full agonists and fewer side reactions than antagonists [37].

However, most D3R partial agonists induce pharmacological sequestration and desensitization significantly. For example, pharmacological sequestration induced by 10 μM quinpirole (a FDA approved drug) or 7-OH-DPAT [7-hydroxy-2-(N, N-di-n-propylamino)tetralin] can achieve 50% or 70%, respectively (Figure 1) [50]. It is gratifying that some new D3R partial agonists without sequestration inducing activity have been developed. SK608 and its analogues have a biased signaling property via the G-protein-coupled receptors but do not result in D3R desensitization [48,49]. On the contrary, SK608 causes D3R endocytosis (Figure 1) in a time-dependent and dose-dependent manner [49]. G-protein-coupled receptor kinase 2 (GRK2) and clathrin/dynamin I/II are the irreplaceable mediators in the SK608-derived D3R endocytosis, but β-arrestin and GRK-interacting protein 1 (GIT1) are not related with this process [49]. These data suggested that SK608-induced D3R internalization is very close to the type II internalization reported among all G-protein-coupled receptors [49]. The 3 μM dopamine treatment at 37 °C for 1 h resulted in a 20% decrease in the D3R protein steady-state level [49]. Thus, SK608 may be an ideal adjuvant drug for addiction rehabilitation, as it can stimulate D3R during substance withdrawal, alleviating the abstinence syndrome, and, in the long run, it may promote D3R degradation, down-regulate the D3R protein steady-state level, and help to achieve complete substance abuse rehabilitation.

## 4. Drugs for Serotoninergic Neurons

While extracellular dopamine accumulation is sufficient to drive compulsion to substances, psychostimulants, like cocaine, also boost extracellular 5-hydroxytryptamine (5-HT; serotonin) by repressing its reuptake [62,63]. Cocaine increased the substance-evoked serotonin efflux in the NAc (Figure 1), suggesting that serotonin also plays a vital role in the pathophysiology of addiction [64]. Nevertheless, further increasing serotonin pharmacologically reversed the compulsion to cocaine, which may be explained by the presynaptic inhibition of orbitofrontal cortex-to-dorsal striatum synapses induced by serotonin [65]. A combination therapy containing lorcaserin (serotonin 5-HT2C receptor agonist) and buspirone (5-HT1A receptor partial agonist and D3R antagonist as mentioned above) produced modest declines in cocaine self-administration in rhesus monkeys [66]. Serotonin reuptake inhibitors citalopram and escitalopram induced the internalization and decrease in cellular serotonin transporters (SERTs; Figure 1) [67], and decreased compulsive cocaine seeking in mice [65,68]. A double-blind clinical trial compared a placebo with citalopram treatments on the duration of cocaine withdrawal, and provided some evidence for positive effects on the longest duration of withdrawal and negative urine cocaine screens [69].

However, regulating serotonin has little influence on dopamine homeostasis, which is still the crucial pathological mechanism of substance addiction. Drugs for serotoninergic neurons may only show some auxiliary effects to the substance abusers. Suchting et al. [69] found that neither 20 mg nor 40 mg citalopram achieved the significant threshold for the primary outcomes, and only the 40 mg dose was declared the “winner” in that trial. Furthermore, compared with dopamine, serotonin is more related with prosocial behaviors. The deletion of kappa opioid receptors (KORs) from serotonin neurons, but not from NAc neurons or dopamine neurons, prevented the sociability deficit during the withdrawal of opioids, because, after withdrawal, KORs block the serotonin release in the NAc that usually occurs during social interactions [70]. For alleviating withdrawal symptoms unrelated with prosocial behaviors, serotonin reuptake inhibitors or serotonin receptor agonists may not be ideal drugs.

## 5. Drugs for GABAergic Neurons

Although dopamine plays the key role in substance dependence, some rewarding effects of substances are mediated by other mechanisms independent of dopamine. The NAc integrates both glutamatergic and dopaminergic inputs to regulate the rewarding and destructive properties of psycho-stimulants [71]. Vega-Villar et al. [72] demonstrated that the substance-evoked signaling requires N-methyl-D-aspartic acid (NMDA) receptor (NMDAR)-dependent plasticity within the NAc, revealing a key role of glutamatergic excitatory and γ-aminobutyric acid (GABA)ergic inhibitory neurons in addiction behaviors. Repeated opioid (such as papaverine, morphine, heroin, ketamine, et al.) exposure strengthens synaptic NMDAR activities in the NAc [73]. Then, opioids elicit robust dopamine transients in the NAc through the derepression of dopamine neurons via the NMDAR antagonism (Figure 1) [74,75,76]. On the other hand, the long-range GABAergic projection from the VTA to the ventral NAc shell, but not to the NAc core or dorsal NAc shell, is also engaged in rewarding and reinforcement behaviors [77]. A single ketamine injection to mice induced a dopamine transient in the NAc that was almost the same in magnitude, whereas of a shorter persistence, than that induced by cocaine. But ketamine did not lead to a change in synaptic plasticity that is usually induced by cocaine. Therefore, ketamine may not induce locomotor sensitization or strong substance seeking. The risk of addiction to ketamine may be lower than cocaine [76]. The reactivation of inhibited NMDAR in person with prolonged opioid use can led to a severe abstinence syndrome. A randomized double-blind clinical trial found that magnesium sulfate, a NMDAR antagonist, showed significantly mitigative effects on the opioid withdrawal syndrome [78]. However, the intravenous infusion of MgSO_4_ may not be a feasible therapy to most substance abusers.

Different from the direct binding of opioids with NMDAR, repeated cocaine exposures enhance GABA release in astrocytes through volume-regulated anion channels (VRACs) and promote the tonic repression of GABAergic neurons in VTA, therefore down-regulating their activities and derepressing NAc-projecting dopamine neurons [79]. Rats with a high cocaine-dependence index exhibited enhanced substance seeking behaviors and tonic GABA release in the amygdala [80].

**Figure 2:**
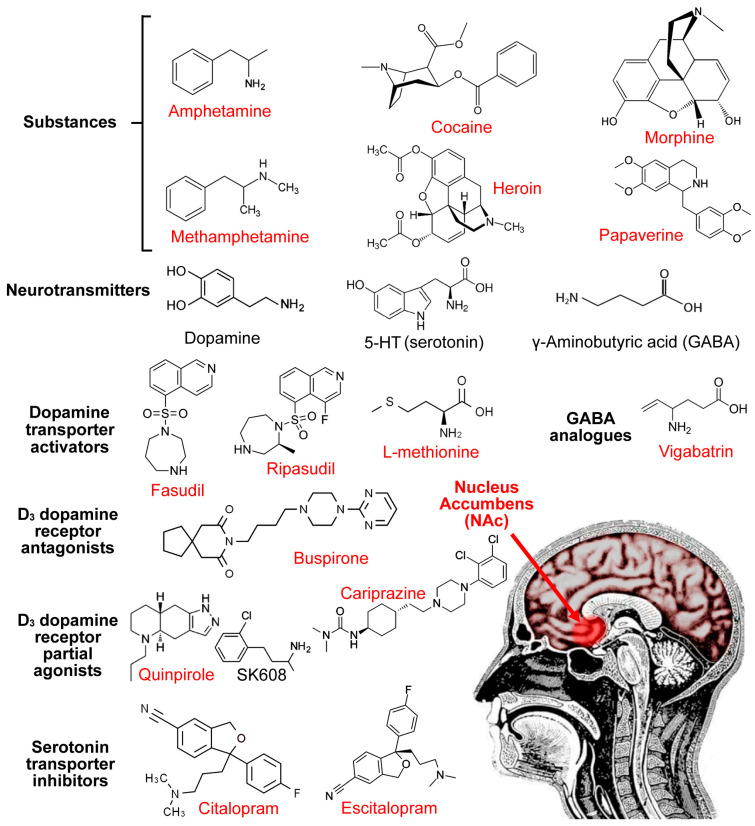
Chemical structures of addiction therapeutic drugs and their blood–brain barrier (BBB) permeability. BBB-penetrable drugs are marked with red color; BBB-impenetrable neurotransmitters and drugs are marked with the black color. Anatomical positions of the nasal cavity and the nucleus accumbens (NAc; indicated by a red arrow) are shown.

It can be inferred that GABA or its analogues could be used for the treatment of substance addiction. But GABA cannot penetrate the blood–brain barrier (BBB), so its lipophilic analogues were developed (Figure 2). A previous study with the rat model demonstrated that gamma-vinyl GABA (vigabatrin), a GABA transaminase antagonist, attenuated the addiction to cocaine (Figure 1) [81]. For alcohol addiction, vigabatrin greatly decreased the number of patients requiring high-dose diazepam over the duration of their alcohol abstinence and was correlated with a decline in adverse effects [82]. New GABA aminotransferase (GABA-AT) inhibitors other than vigabatrin have also been developed. For example, CPP-115 and Compound 5 showed more efficiency in inactivating GABA-AT than vigabatrin and inhibited the dopamine transients in the corpus striatum upon an acute cocaine or nicotine challenge [83].

Cocaine and amphetamine act on DAT directly, while opioids elicit dopamine transients indirectly through modifying GABAergic neurons. Thus, GABA analogues may be less effective for cocaine or amphetamine addiction than for opioid dependence. In a placebo-controlled double-blind clinical trial for treating cocaine addiction, no protocol-defined difference in efficacy between vigabatrin treatments and the placebo was found [84].

## 6. Drugs for Synaptic Plasticity

The hallucinogenic alkaloid ibogaine has been shown in anecdotal and open-label studies to decrease substance abuse in the clinic [85]. Like other psychedelic drugs, the therapeutic effect of ibogaine is long-lasting [86], which may be attributed to its capability to regulate addiction-related neural circuits through the activation of neurotrophic factors, e.g., brain-derived neurotrophic factor (BDNF) and glial-cell-line-derived neurotrophic factor (GDNF) [87]. Nevertheless, many safety concerns have hampered the clinical development of ibogaine including the cytotoxicity, hallucinogenic action, and side effect of inducing cardiac arrhythmias. Recently, a non-toxic and non-hallucinogenic analogue, tabernanthalog (TBG), has been developed. TBG enhanced the structural synaptic plasticity, and decreased heroin and alcohol-seeking behaviors in rats [88].

Besides G-protein-coupled signaling, dopamine also works as a donor for the post-translation modification (dopaminylation) on some enzymes, for example, histone H3 glutamine 5 dopaminylation (H3Q5dop) [89]. In rats, after cocaine withdrawal, a rapid H3Q5dop increase was observed in the NAc. The inhibition of H3Q5dop in NAc after withdrawal decreased the relapse-like behaviors and reversed cocaine-derived transcription changes [90]. Besides H3Q5dop, DNA methylation also has an influence on locomotor-activating and on substance addiction [25,91,92]. All of this research suggests that epigenetic mechanisms, e.g., histone modification, may be involved in the responsiveness to substance abuse. Whether ibogaine and its analogues affect the neuro-plasticity related epigenetic signatures needs further investigations.

## 7. Drugs for Neuroinflammation

The neuro-immune responses to substance abuse are characterized by the proliferation and functional and morphological alters of astrocytes and microglia. The microglia respond directly to substance-induced neuronal injuries and are associated with the activation of cytokine and chemokine signals, putatively resulting from substance-induced damages to the BBB [14,93]. Microglial activation causes the cell migration to the damage sites, and the releasing of inflammatory factors, such as tumor necrosis factor-α (TNF-α) and interleukins [94]. Astrocytes also produce inflammatory factors upon BBB damage. Furthermore, excess dopamine may activate DA receptors on glial cells and extend pro-inflammatory signals by a further releasing of chemokines and cytokines [95]. Recently, Zhu et al. [96] found that a significant increase in fragile-like regulatory T cells and increased interferon-g expression were positively associated with the opioid abstinence score in mice. They indicated that opioids enhanced the neuron-derived C-C motif chemokine ligand 2 (Ccl2), caused BBB injuries, and induced peripherally regulatory T-cell infiltration into NAc, also suggesting a role of neuroinflammation in the withdrawal symptoms.

Accordingly, anti-inflammatory drugs have been suggested to treat substance abuse (Table 1) [95]. For example, rosuvastatin not only delayed, but also partially reversed, the tolerance to morphine-induced analgesia in rats by attenuating the releasing of pro-inflammatory cytokines in the lumbar spinal cord [97]. Repeated morphine administration activated the astrocytes, while rosuvastation suppressed this activation [98]. Rosuvastatin reversed the morphine tolerance also by inhibiting Rho GTPases activation [17,97,98,99,100]. However, a clinical trial of simvastatin on smoking cessation found that a 3-month simvastatin administration did not improve smoking cessation significantly compared with the placebo group [101]. Human clinical evidence of rosuvastatin on substance addiction is still lacking. Ibudilast attenuated methamphetamine addiction in mice [102], while a high-dose ibudilast (100 mg) treatment reduced methamphetamine self-administration and craving in humans [103]. Minocycline ameliorated the cognitive impairment and synaptic dysfunction induced by methamphetamine in mice [104], while minocycline reduced the amphetamine-induced subjective rewarding effects in humans [105]. Pioglitazone (a peroxisome proliferator-activated receptor PPAR-γ agonist) attenuated behavioral sensitization during the withdrawal of methamphetamine in mice [106], while, in a clinical trial, pioglitazone reduced the craving for cocaine and enhanced white-matter integrity in cocaine abusers [107]. However, another report showed that pioglitazone was marginally effective in reducing the reinforcing effects of nicotine and the nicotine-seeking behaviors [108].

## 8. BBB-Penetrable Drug Delivery Methods

Many FDA-approved drugs for substance addiction have shown little to no evidence of addiction in human clinical trials (Table 1). Additionally, some of these drugs have a limited ability to cross the blood–brain barrier (BBB), which limits their effectiveness. Dopamine also cannot penetrate the BBB, so it may be inferred that its analogue SK608 cannot either (Figure 2). Utilizing nano-dimensional vehicles with vast surface areas can help overcome the difficulties in penetration across the complicated physiology of the BBB [109,110]. Alternatively, intranasal administration is a non-invasive method that bypasses the BBB to allow the direct access of drugs to the central nervous system, which has been showing promising results recently [111,112,113]. The entry of drugs into the brain via the nasal cavity could be divided into direct paths and indirect paths. In the direct paths, drugs may bypass the BBB by entering the neurocytes through either the trigeminal or olfactory nerves in the nasal cavity. This process would take place either by extracellular or intracellular pathways, i.e., the transcellular pathway and paracellular pathway, while, in the indirect paths, drugs may be absorbed into the systemic circulation through capillaries present in the nasal mucosa, and then gain access to neurocytes by crossing the BBB [111]. Anatomically, the nasal cavity is close to the nucleus accumbens (Figure 2). Therefore, SK608 nebulization therapy or a nasal spray might show a promising result for substance abusers.

The eye, an anatomical extension of the central nervous system (CNS), exhibits many molecular and cellular parallels to the brain. Recently, Yin et al. [114] studied immune responses to herpes simplex virus in the brain, and observed that intravitreal immunization protects mice against an intracranial viral challenge. These results revealed a shared lymphatic circuit able to mount a unified immune response between the posterior eye and the brain, highlighting an understudied immunological feature of the eye and opening up the potential for new therapeutic strategies in ocular and CNS diseases, maybe including substance addiction.

## 9. Conclusions

D3R partial agonists may mitigate both the substance dependence and withdrawal symptoms. Cariprazine is the only FDA-approved D3R agonist showing significantly beneficial effects to substance addiction (Table 1). However, its clinical trials are still needed. Most D3R partial agonists induce pharmacological sequestration and desensitization. SK608 may be the most promising drug without sequestration-inducing activities, but the research on it is currently limited at the cellular level. Animal model experiments, human clinical trials, and toxicological and pharmacological trials are still required. SK608 cannot penetrate the BBB. Therefore, SK608 nebulization therapy or a nasal spray is proposed. Alternatively, BBB-penetrable D3R agonists without sequestration-inducing activities may be developed. Drugs for serotoninergic neurons may show some auxiliary effects to the substance abusers, such as a high dose of citalopram (Table 1). New serotonin reuptake inhibitors with a higher affinity and selectivity should be developed. GABA transaminase inhibitors, e.g., vigabatrin, may treat opioid dependence, but showed less efficiency for cocaine or amphetamine addiction (Table 1). Non-hallucinogenic analogues of ibogaine are also promising drugs, since they promote structural neural plasticity after substance challenges. Neuroinflammation may play only a minor role in substance addiction, although some anti-inflammatory drugs showed beneficial effects. Among FDA-approved drugs, L-methionine may be the most promising one for clinical uses with significant therapeutic effects but fewer side effects.

It is intriguing that substances like methadone, buprenorphine, naltrexone, or disulfiram, and the cellular pathways modulated by them have not been explored. Nicotine and alcohol addictions also represent serious problems for contemporary society. Rehabilitation drugs to treat addiction to nicotine, alcohol, or new types of substances still need to be developed.

## Figures and Tables

**Table 1:** FDA-approved drugs for substance addiction.

Drug Name (Administration Form)	Therapeutic Mechanisms	Evidence in Animal Experiments of Addiction	Evidence from Human Clinical Trials to Addiction	Refs.
L-methionine (oral or injection)	Down-regulating *Vav2* gene and influencing DNA methylation in NAc	Inhibiting cocaine dependence in mice and rats	None	[23,24,25]
Fasudil/Ripasudil (oral or injection)	ROCK inhibitor; promoting DAT recycling	Ameliorating spatial learning and memory disorders induced by smoking in mice	None	[31]
Buspirone (oral)	D3R antagonist; 5-HT1A receptor partial agonist	Abolishing cocaine and alcohol primed reinstatement in mice, dogs, and monkeys	Ineffectiveness to relapse to cocaine	[44,45,46,47]
Quinpirole (injection)	D3R partial agonist	No significant effect on the place preference induced by cocaine or morphine in rats	None	[60]
Cariprazine (oral or injection)	D3R partial agonist	Decreasing cocaine self-administration in rats	None	[61]
Citalopram/Escitalopram (oral or injection)	Serotonin reuptake inhibitor	Decreasing compulsive cocaine self-administration in mice	A high dose showed positive effects on the longest duration of cocaine abstinence	[65,68,69]
Vigabatrin (oral or injection)	GABA transaminase inhibitor	Attenuating the acute rewarding effects of cocaine in rats	Alleviating alcohol addiction; ineffectiveness for cocaine dependence	[81,82,84]
Rosuvastatin/Simvastatin (oral)	Anti-neuro-inflammation; inhibiting Rho GTPases activation	Reversing the tolerance to morphine-induced analgesia in rats	Ineffectiveness for smoking cessation	[97,101]
Ibudilast (oral)	Anti-inflammatory drug	Attenuating methamphetamine addiction in mice	A high dose reduced methamphetamine self-administration and craving	[102,103]
Minocycline (oral or injection)	Anti-inflammatory drug	Ameliorating cognitive impairment induced by methamphetamine in mice	Reducing amphetamine induced subjective rewarding effects	[104,105]
Pioglitazone (oral)	Anti-inflammatory drug	Attenuating behavioral sensitization during the withdrawal of methamphetamine in mice	Reducing craving for cocaine; showing marginal effects to nicotine addiction	[106,107,108]

## Data Availability

Not applicable.

## Abbreviations

5-HT	5-hydroxytryptamine
BBB	blood–brain barrier
BDNF	brain-derived neurotrophic factor
Ccl2	C-C motif chemokine ligand 2
D2R	D_2_ dopamine receptor
D3R	D_3_ dopamine receptor
DAT	dopamine transporter
FDA	Food and Drug Administration
GABA	γ-aminobutyric acid
GABA-AT	GABA aminotransferase
GDNF	glial cell line-derived neurotrophic factor
H3Q5dop	histone H3 glutamine 5 dopaminylation
KOR	kappa opioid receptor
NAc	nucleus accumbens
NMDA	N-methyl-D-aspartic acid
NMDAR	NMDA receptor
PET	positron emission tomography
PPAR	peroxisome proliferator-activated receptor
ROCK	Rho-associated protein kinase
SERT	serotonin transporter
TBG	tabernanthalog
TNF-α	tumor necrosis factor-α
VRAC	volume-regulated anion channel
VTA	ventral tegmental area

## References

[1] World Drug Report 2023. https://www.unodc.org/unodc/en/data-and-analysis/world-drug-report-2023.html.

[2] Hagemeier N.E. (2018). Introduction to the opioid epidemic: The economic burden on the healthcare system and impact on quality of life. Am. J. Manag. Care.

[3] Stewart S.A., Copeland A.L., Cherry K.E. (2023). Risk factors for substance use across the lifespan. J. Genet. Psychol..

[4] Merikangas K.R., Kalaydjian A. (2007). Magnitude and impact of comorbidity of mental disorders from epidemiologic surveys. Curr. Opin. Psychiatry.

[5] Verdejo-Garcia A., Lorenzetti V., Manning V., Piercy H., Bruno R., Hester R., Pennington D., Tolomeo S., Arunogiri S., Bates M.E. (2019). A roadmap for integrating neuroscience into addiction treatment: A consensus of the neuroscience interest group of the International Society of Addiction Medicine. Front. Psychiatry.

[6] Frawley P.J., Smith J.W. (1992). One-year follow-up after multimodal inpatient treatment for cocaine and methamphetamine dependencies. J. Subst. Abuse Treat..

[7] Kassani A., Niazi M., Hassanzadeh J., Menati R. (2015). Survival analysis of drug abuse relapse in addiction treatment centers. Int. J. High Risk Behav. Addict..

[8] Monroe S.C., Radke A.K. (2023). Opioid withdrawal: Role in addiction and neural mechanisms. Psychopharmacology.

[9] Tabanelli R., Brogi S., Calderone V. (2023). Targeting opioid receptors in addiction and drug withdrawal: Where are we going?. Int. J. Mol. Sci..

[10] Ciucă Anghel D.M., Nițescu G.V., Tiron A.T., Guțu C.M., Baconi D.L. (2023). Understanding the mechanisms of action and effects of drugs of abuse. Molecules.

[11] Padhan M., Maiti R., Mohapatra D., Mishra B.R. (2023). Efficacy and safety of tramadol in the treatment of opioid withdrawal: A meta-analysis of randomized controlled trials. Addict. Behav..

[12] Volkow N.D., Morales M. (2015). The brain on drugs: From reward to addiction. Cell.

[13] Bayassi-Jakowicka M., Lietzau G., Czuba E., Steliga A., Waśkow M., Kowiański P. (2021). Neuroplasticity and multilevel system of connections determine the integrative role of nucleus accumbens in the brain reward system. Int. J. Mol. Sci..

[14] Karimi-Haghighi S., Chavoshinezhad S., Mozafari R., Noorbakhsh F., Borhani-Haghighi A., Haghparast A. (2023). Neuroinflammatory response in reward-associated psychostimulants and opioids: A review. Cell. Mol. Neurobiol..

[15] Rezayof A., Ghasemzadeh Z., Sahafi O.H. (2023). Addictive drugs modify neurogenesis, synaptogenesis and synaptic plasticity to impair memory formation through neurotransmitter imbalances and signaling dysfunction. Neurochem. Int..

[16] Liu X., Wang F., Le Q., Ma L. (2023). Cellular and molecular basis of drug addiction: The role of neuronal ensembles in addiction. Curr. Opin. Neurobiol..

[17] Ru Q., Wang Y., Zhou E., Chen L., Wu Y. (2023). The potential therapeutic roles of Rho GTPases in substance dependence. Front. Mol. Neurosci..

[18] Støier J.F., Konomi-Pilkati A., Apuschkin M., Herborg F., Gether U. (2023). Amphetamine-induced reverse transport of dopamine does not require cytosolic Ca^2+^. J. Biol. Chem..

[19] Saunders C., Galli A. (2015). Insights in how amphetamine ROCKs (Rho-associated containing kinase) membrane protein trafficking. Proc. Natl. Acad. Sci. USA.

[20] Wheeler D.S., Underhill S.M., Stolz D.B., Murdoch G.H., Thiels E., Romero G., Amara S.G. (2015). Amphetamine activates Rho GTPase signaling to mediate dopamine transporter internalization and acute behavioral effects of amphetamine. Proc. Natl. Acad. Sci. USA.

[21] Zhu S., Zhao C., Wu Y., Yang Q., Shao A., Wang T., Wu J., Yin Y., Li Y., Hou J. (2015). Identification of a Vav2-dependent mechanism for GDNF/Ret control of mesolimbic DAT trafficking. Nat. Neurosci..

[22] Refai O., Aggarwal S., Cheng M.H., Gichi Z., Salvino J.M., Bahar I., Blakely R.D., Mortensen O.V. (2022). Allosteric modulator KM822 attenuates behavioral actions of amphetamine in *Caenorhabditis elegans* through interactions with the dopamine transporter DAT-1. Mol. Pharmacol..

[23] Zhang K., Ji G., Zhao M., Wang Y. (2021). Candidate l-methionine target piRNA regulatory networks analysis response to cocaine-conditioned place preference in mice. Brain Behav..

[24] Wang Y., Yang L., Zhou H., Zhang K., Zhao M. (2023). Identification of miRNA-mediated gene regulatory networks in L-methionine exposure counteracts cocaine-conditioned place preference in mice. Front. Genet..

[25] Wright K.N., Hollis F., Duclot F., Dossat A.M., Strong C.E., Francis T.C., Mercer R., Feng J., Dietz D.M., Lobo M.K. (2015). Methyl supplementation attenuates cocaine-seeking behaviors and cocaine-induced c-Fos activation in a DNA methylation-dependent manner. J. Neurosci..

[26] Gabriel L.R., Wu S., Kearney P., Bellvé K.D., Standley C., Fogarty K.E., Melikian H.E. (2013). Dopamine transporter endocytic trafficking in striatal dopaminergic neurons: Differential dependence on dynamin and the actin cytoskeleton. J. Neurosci..

[27] Swanson A.M., DePoy L.M., Gourley S.L. (2017). Inhibiting Rho kinase promotes goal-directed decision making and blocks habitual responding for cocaine. Nat. Commun..

[28] Bagalkot T., Sorkin A. (2024). Amphetamine induces sex-dependent loss of the striatal dopamine transporter in sensitized mice. eNeuro.

[29] Tatenhorst L., Eckermann K., Dambeck V., Fonseca-Ornelas L., Walle H., Lopes da Fonseca T., Koch J.C., Becker S., Tönges L., Bähr M. (2016). Fasudil attenuates aggregation of alpha-synuclein in models of Parkinson’s disease. Acta Neuropathol. Commun..

[30] Abedi F., Hayes A.W., Reiter R., Karimi G. (2020). Acute lung injury: The therapeutic role of Rho kinase inhibitors. Pharmacol. Res..

[31] Xueyang D., Zhanqiang M., Chunhua M., Kun H. (2016). Fasudil, an inhibitor of Rho-associated coiled-coil kinase, improves cognitive impairments induced by smoke exposure. Oncotarget.

[32] Galaj E., Ananthan S., Saliba M., Ranaldi R. (2014). The effects of the novel DA D3 receptor antagonist SR 21502 on cocaine reward, cocaine seeking and cocaine-induced locomotor activity in rats. Psychopharmacology.

[33] Pribiag H., Shin S., Wang E.H., Sun F., Datta P., Okamoto A., Guss H., Jain A., Wang X.Y., De Freitas B. (2021). Ventral pallidum DRD3 potentiates a pallido-habenular circuit driving accumbal dopamine release and cocaine seeking. Neuron.

[34] Segal D.M., Moraes C.T., Mash D.C. (1997). Up-regulation of D3 dopamine receptor mRNA in the nucleus accumbens of human cocaine fatalities. Brain Res. Mol. Brain Res..

[35] Matuskey D., Gallezot J.D., Pittman B., Williams W., Wanyiri J., Gaiser E., Lee D.E., Hannestad J., Lim K., Zheng M.Q. (2014). Dopamine D_3_ receptor alterations in cocaine-dependent humans imaged with [¹¹C](+)PHNO. Drug Alcohol Depend..

[36] Czoty P.W., Tryhus A.M., Solingapuram Sai K.K., Nader S.H., Epperly P.M. (2023). Association of dopamine D2-like and D3 receptor function with initial sensitivity to cocaine reinforcement in male rhesus monkeys. Brain Res..

[37] Cortés A., Moreno E., Rodríguez-Ruiz M., Canela E.I., Casadó V. (2016). Targeting the dopamine D3 receptor: An overview of drug design strategies. Expert Opin. Drug Discov..

[38] Kumar V., Bonifazi A., Ellenberger M.P., Keck T.M., Pommier E., Rais R., Slusher B.S., Gardner E., You Z.B., Xi Z.X. (2016). Highly selective dopamine D3 receptor (D3R) antagonists and partial agonists based on eticlopride and the D3R crystal structure: New leads for opioid dependence treatment. J. Med. Chem..

[39] Manvich D.F., Petko A.K., Branco R.C., Foster S.L., Porter-Stransky K.A., Stout K.A., Newman A.H., Miller G.W., Paladini C.A., Weinshenker D. (2019). Selective D2 and D3 receptor antagonists oppositely modulate cocaine responses in mice via distinct postsynaptic mechanisms in nucleus accumbens. Neuropsychopharmacology.

[40] Xi Z.X., Li X., Li J., Peng X.Q., Song R., Gaál J., Gardner E.L. (2013). Blockade of dopamine D3 receptors in the nucleus accumbens and central amygdale inhibits incubation of cocaine craving in rats. Addict. Biol..

[41] Botz-Zapp C.A., Foster S.L., Pulley D.M., Hempel B., Bi G.H., Xi Z.X., Newman A.H., Weinshenker D., Manvich D.F. (2021). Effects of the selective dopamine D3 receptor antagonist PG01037 on morphine-induced hyperactivity and antinociception in mice. Behav. Brain Res..

[42] Newman A.H., Blaylock B.L., Nader M.A., Bergman J., Sibley D.R., Skolnick P. (2012). Medication discovery for addiction: Translating the dopamine D3 receptor hypothesis. Biochem. Pharmacol..

[43] Leggio G.M., Bucolo C., Platania C.B., Salomone S., Drago F. (2016). Current drug treatments targeting dopamine D3 receptor. Pharmacol. Ther..

[44] Bergman J., Roof R.A., Furman C.A., Conroy J.L., Mello N.K., Sibley D.R., Skolnick P. (2013). Modification of cocaine self-administration by buspirone (buspar^®^): Potential involvement of D3 and D4 dopamine receptors. Int. J. Neuropsychopharmacol..

[45] Leggio G.M., Camillieri G., Platania C.B., Castorina A., Marrazzo G., Torrisi S.A., Nona C.N., D’Agata V., Nobrega J., Stark H. (2014). Dopamine D3 receptor is necessary for ethanol consumption: An approach with buspirone. Neuropsychopharmacology.

[46] Appel N.M., Li S.H., Holmes T.H., Acri J.B. (2015). Dopamine D3 receptor antagonist (GSK598809) potentiates the hypertensive effects of cocaine in conscious, freely-moving dogs. J. Pharmacol. Exp. Ther..

[47] Winhusen T.M., Kropp F., Lindblad R., Douaihy A., Haynes L., Hodgkins C., Chartier K., Kampman K.M., Sharma G., Lewis D.F. (2014). Multisite, randomized, double-blind, placebo-controlled pilot clinical trial to evaluate the efficacy of buspirone as a relapse-prevention treatment for cocaine dependence. J. Clin. Psychiatry.

[48] Xu W., Wang X., Tocker A.M., Huang P., Reith M.E., Liu-Chen L.Y., Smith A.B., Kortagere S. (2017). Functional characterization of a novel series of biased signaling dopamine D3 receptor agonists. ACS Chem. Neurosci..

[49] Xu W., Reith M.E.A., Liu-Chen L.Y., Kortagere S. (2019). Biased signaling agonist of dopamine D3 receptor induces receptor internalization independent of β-arrestin recruitment. Pharmacol. Res..

[50] Min C., Zheng M., Zhang X., Caron M.G., Kim K.M. (2013). Novel roles for β-arrestins in the regulation of pharmacological sequestration to predict agonist-induced desensitization of dopamine D3 receptors. Br. J. Pharmacol..

[51] Slosky L.M., Bai Y., Toth K., Ray C., Rochelle L.K., Badea A., Chandrasekhar R., Pogorelov V.M., Abraham D.M., Atluri N. (2020). β-arrestin-biased allosteric modulator of NTSR1 selectively attenuates addictive behaviors. Cell.

[52] Huang B., Li Y., Cheng D., He G., Liu X., Ma L. (2018). β-Arrestin-biased β-adrenergic signaling promotes extinction learning of cocaine reward memory. Sci. Signal..

[53] Zhang X., Sun N., Zheng M., Kim K.M. (2016). Clathrin-mediated endocytosis is responsible for the lysosomal degradation of dopamine D3 receptor. Biochem. Biophys. Res. Commun..

[54] Keck T.M., John W.S., Czoty P.W., Nader M.A., Newman A.H. (2015). Identifying medication targets for psychostimulant addiction: Unravelling the dopamine D3 receptor hypothesis. J. Med. Chem..

[55] Kiss B., Krámos B., Laszlovszky I. (2022). Potential mechanisms for why not all antipsychotics are able to occupy dopamine D3 receptors in the brain in vivo. Front. Psychiatry.

[56] Moritz A.E., Free R.B., Weiner W.S., Akano E.O., Gandhi D., Abramyan A., Keck T.M., Ferrer M., Hu X., Southall N. (2020). Discovery, optimization, and characterization of ML417: A novel and highly selective D3 dopamine receptor agonist. J. Med. Chem..

[57] Kuzhikandathil E.V., Kortagere S. (2012). Identification and characterization of a novel class of atypical dopamine receptor agonists. Pharm. Res..

[58] Galaj E., Newman A.H., Xi Z.X. (2020). Dopamine D3 receptor-based medication development for the treatment of opioid use disorder: Rationale, progress, and challenges. Neurosci. Biobehav. Rev..

[59] Kiss B., Laszlovszky I., Krámos B., Visegrády A., Bobok A., Lévay G., Lendvai B., Román V. (2021). Neuronal dopamine D3 receptors: Translational implications for preclinical research and CNS disorders. Biomolecules.

[60] Kivastik T., Vuorikallas K., Piepponen T.P., Zharkovsky A., Ahtee L. (1996). Morphine- and cocaine-induced conditioned place preference: Effects of quinpirole and preclamol. Pharmacol. Biochem. Behav..

[61] Gogarnoiu E.S., Vogt C.D., Sanchez J., Bonifazi A., Saab E., Shaik A.B., Soler-Cedeño O., Bi G.H., Klein B., Xi Z.X. (2023). Dopamine D3/D2 receptor ligands based on cariprazine for the treatment of psychostimulant use disorders that may be dual diagnosed with affective disorders. J. Med. Chem..

[62] Simmler L.D., Anacker A.M.J., Levin M.H., Vaswani N.M., Gresch P.J., Nackenoff A.G., Anastasio N.C., Stutz S.J., Cunningham K.A., Wang J. (2017). Blockade of the 5-HT transporter contributes to the behavioural, neuronal and molecular effects of cocaine. Br. J. Pharmacol..

[63] Simmler L.D., Blakely R.D. (2019). The SERT Met172 mouse: An engineered model to elucidate the contributions of serotonin signaling to cocaine action. ACS Chem. Neurosci..

[64] Yuen J., Goyal A., Rusheen A.E., Kouzani A.Z., Berk M., Kim J.H., Tye S.J., Blaha C.D., Bennet K.E., Lee K.H. (2022). Cocaine increases stimulation-evoked serotonin efflux in the nucleus accumbens. J. Neurophysiol..

[65] Li Y., Simmler L.D., Van Zessen R., Flakowski J., Wan J.X., Deng F., Li Y.L., Nautiyal K.M., Pascoli V., Lüscher C. (2021). Synaptic mechanism underlying serotonin modulation of transition to cocaine addiction. Science.

[66] Collins G.T., France C.P. (2018). Effects of lorcaserin and buspirone, administered alone and as a mixture, on cocaine self-administration in male and female rhesus monkeys. Exp. Clin. Psychopharmacol..

[67] Brown C.R., Foster J.D. (2023). Palmitoylation regulates human serotonin transporter activity, trafficking, and expression and is modulated by escitalopram. ACS Chem. Neurosci..

[68] Chung S., Kim H.J., Kim H.J., Choi S.H., Kim J.W., Kim J.M., Shin K.H. (2014). Effect of desipramine and citalopram treatment on forced swimming test-induced changes in cocaine- and amphetamine-regulated transcript (CART) immunoreactivity in mice. Neurochem. Res..

[69] Suchting R., Green C.E., de Dios C., Vincent J., Moeller F.G., Lane S.D., Schmitz J.M. (2021). Citalopram for treatment of cocaine use disorder: A Bayesian drop-the-loser randomized clinical trial. Drug Alcohol Depend..

[70] Pomrenze M.B., Cardozo Pinto D.F., Neumann P.A., Llorach P., Tucciarone J.M., Morishita W., Eshel N., Heifets B.D., Malenka R.C. (2022). Modulation of 5-HT release by dynorphin mediates social deficits during opioid withdrawal. Neuron.

[71] Volkow N.D., Michaelides M., Baler R. (2019). The neuroscience of drug reward and addiction. Physiol. Rev..

[72] Vega-Villar M., Horvitz J.C., Nicola S.M. (2019). NMDA receptor-dependent plasticity in the nucleus accumbens connects reward-predictive cues to approach responses. Nat. Commun..

[73] Jin D., Chen H., Chen S.R., Pan H.L. (2023). α2δ-1 protein drives opioid-induced conditioned reward and synaptic NMDA receptor hyperactivity in the nucleus accumbens. J. Neurochem..

[74] Anderson E.M., Reeves T., Kapernaros K., Neubert J.K., Caudle R.M. (2015). Phosphorylation of the N-methyl-d-aspartate receptor is increased in the nucleus accumbens during both acute and extended morphine withdrawal. J. Pharmacol. Exp. Ther..

[75] Kim A., Gu S.M., Lee H., Kim D.E., Hong J.T., Yun J., Cha H.J. (2023). Prenatal ketamine exposure impairs prepulse inhibition via arginine vasopressin receptor 1A-mediated GABAergic neuronal dysfunction in the striatum. Biomed. Pharmacother..

[76] Simmler L.D., Li Y., Hadjas L.C., Hiver A., van Zessen R., Lüscher C. (2022). Dual action of ketamine confines addiction liability. Nature.

[77] Al-Hasani R., Gowrishankar R., Schmitz G.P., Pedersen C.E., Marcus D.J., Shirley S.E., Hobbs T.E., Elerding A.J., Renaud S.J., Jing M. (2021). Ventral tegmental area GABAergic inhibition of cholinergic interneurons in the ventral nucleus accumbens shell promotes reward reinforcement. Nat. Neurosci..

[78] Moshiri M., Chaeideh B., Ebrahimi M., Dadpour B., Ghodsi A., Haghighizadeh A., Etemad L. (2024). Buprenorphine induced opioid withdrawal syndrome relieved by adjunctive Magnesium: A clinical trial. J. Subst. Use Addict. Treat..

[79] Yang J., Chen J., Liu Y., Chen K.H., Baraban J.M., Qiu Z. (2023). Ventral tegmental area astrocytes modulate cocaine reward by tonically releasing GABA. Neuron.

[80] Zhou J.L., de Guglielmo G., Ho A.J., Kallupi M., Pokhrel N., Li H.R., Chitre A.S., Munro D., Mohammadi P., Carrette L.L.G. (2023). Single-nucleus genomics in outbred rats with divergent cocaine addiction-like behaviors reveals changes in amygdala GABAergic inhibition. Nat. Neurosci..

[81] Peng X.Q., Li X., Gilbert J.G., Pak A.C., Ashby Jr C.R., Brodie J.D., Dewey S.L., Gardner E.L., Xi Z.X. (2008). Gamma-vinyl GABA inhibits cocaine-triggered reinstatement of drug-seeking behavior in rats by a non-dopaminergic mechanism. Drug Alcohol Depend..

[82] Williams J., Collins L., Norman A., O’Neill H., Lloyd-Jones M., Ogden E., Bonomo Y., Pastor A. (2023). A placebo-controlled randomized trial of vigabatrin in the management of acute alcohol withdrawal. Alcohol Alcohol..

[83] Juncosa J.I., Takaya K., Le H.V., Moschitto M.J., Weerawarna P.M., Mascarenhas R., Liu D., Dewey S.L., Silverman R.B. (2018). Design and mechanism of (S)-3-amino-4-(difluoromethylenyl)cyclopent-1-ene-1-carboxylic acid, a highly potent γ-aminobutyric acid aminotransferase inactivator for the treatment of addiction. J. Am. Chem. Soc..

[84] Somoza E.C., Winship D., Gorodetzky C.W., Lewis D., Ciraulo D.A., Galloway G.P., Segal S.D., Sheehan M., Roache J.D., Bickel W.K. (2013). A multisite, double-blind, placebo-controlled clinical trial to evaluate the safety and efficacy of vigabatrin for treating cocaine dependence. JAMA Psychiatry.

[85] Wasko M.J., Witt-Enderby P.A., Surratt C.K. (2018). DARK classics in chemical neuroscience: Ibogaine. ACS Chem. Neurosci..

[86] Noller G.E., Frampton C.M., Yazar-Klosinski B. (2018). Ibogaine treatment outcomes for opioid dependence from a twelve-month follow-up observational study. Am. J. Drug Alcohol Abuse.

[87] Marton S., González B., Rodríguez-Bottero S., Miquel E., Martínez-Palma L., Pazos M., Prieto J.P., Rodríguez P., Sames D., Seoane G. (2019). Ibogaine administration modifies GDNF and BDNF expression in brain regions involved in mesocorticolimbic and nigral dopaminergic circuits. Front. Pharmacol..

[88] Cameron L.P., Tombari R.J., Lu J., Pell A.J., Hurley Z.Q., Ehinger Y., Vargas M.V., McCarroll M.N., Taylor J.C., Myers-Turnbull D. (2021). A non-hallucinogenic psychedelic analogue with therapeutic potential. Nature.

[89] Lepack A.E., Werner C.T., Stewart A.F., Fulton S.L., Zhong P., Farrelly L.A., Smith A.C.W., Ramakrishnan A., Lyu Y., Bastle R.M. (2020). Dopaminylation of histone H3 in ventral tegmental area regulates cocaine seeking. Science.

[90] Stewart A.F., Lepack A.E., Fulton S.L., Safovich P., Maze I. (2023). Histone H3 dopaminylation in nucleus accumbens, but not medial prefrontal cortex, contributes to cocaine-seeking following prolonged abstinence. Mol. Cell. Neurosci..

[91] Vaillancourt K., Chen G.G., Fiori L., Maussion G., Yerko V., Théroux J.F., Ernst C., Labonté B., Calipari E., Nestler E.J. (2021). Methylation of the tyrosine hydroxylase gene is dysregulated by cocaine dependence in the human striatum. iScience.

[92] Poisel E., Zillich L., Streit F., Frank J., Friske M.M., Foo J.C., Mechawar N., Turecki G., Hansson A.C., Nöthen M.M. (2023). DNA methylation in cocaine use disorder-An epigenome-wide approach in the human prefrontal cortex. Front. Psychiatry.

[93] Lacagnina M.J., Rivera P.D., Bilbo S.D. (2017). Glial and neuroimmune mechanisms as critical modulators of drug use and abuse. Neuropsychopharmacology.

[94] Beardsley P.M., Hauser K.F. (2014). Glial modulators as potential treatments of psychostimulant abuse. Adv. Pharmacol..

[95] Correia C., Romieu P., Olmstead M.C., Befort K. (2020). Can cocaine-induced neuroinflammation explain maladaptive cocaine-associated memories?. Neurosci. Biobehav. Rev..

[96] Zhu Y., Yan P., Wang R., Lai J., Tang H., Xiao X., Yu R., Bao X., Zhu F., Wang K. (2023). Opioid-induced fragile-like regulatory T cells contribute to withdrawal. Cell.

[97] Li Y., Shu Y., Ji Q., Liu J., He X., Li W. (2015). Attenuation of morphine analgesic tolerance by rosuvastatin in naïve and morphine tolerance rats. Inflammation.

[98] Kim M., Nozu F., Kusama K., Imawari M. (2006). Cholecystokinin stimulates the recruitment of the Src-RhoA-phosphoinositide 3-kinase pathway by Vav-2 downstream of G(alpha13) in pancreatic acini. Biochem. Biophys. Res. Commun..

[99] Bulhak A., Roy J., Hedin U., Sjöquist P.O., Pernow J. (2007). Cardioprotective effect of rosuvastatin in vivo is dependent on inhibition of geranylgeranyl pyrophosphate and altered RhoA membrane translocation. Am. J. Physiol. Heart Circ. Physiol..

[100] Wingard C.J., Moukdar F., Prasad R.Y., Cathey B.L., Wilkinson L. (2009). Reversal of voltage-dependent erectile responses in the Zucker obese-diabetic rat by rosuvastatin-altered RhoA/Rho-kinase signaling. J. Sex Med..

[101] Ingrand I., Solinas M., Ingrand P., Dugast E., Saulnier P.J., Pérault-Pochat M.C., Lafay-Chebassier C. (2018). Lack of effects of simvastatin on smoking cessation in humans: A double-blind, randomized, placebo-controlled clinical study. Sci. Rep..

[102] Loftis J.M., Ramani S., Firsick E.J., Hudson R., Le-Cook A., Murnane K.S., Vandenbark A., Shirley R.L. (2023). Immunotherapeutic treatment of inflammation in mice exposed to methamphetamine. Front. Psychiatry.

[103] Worley M.J., Heinzerling K.G., Roche D.J., Shoptaw S. (2016). Ibudilast attenuates subjective effects of methamphetamine in a placebo-controlled inpatient study. Drug Alcohol Depend..

[104] Kusui Y., Izuo N., Tokuhara R., Asano T., Nitta A. (2024). Neuronal activation of nucleus accumbens by local methamphetamine administration induces cognitive impairment through microglial inflammation in mice. J. Pharmacol. Sci..

[105] Sofuoglu M., Mooney M., Kosten A.T., Waters K. (2011). Hashimoto, Minocycline attenuates subjective rewarding effects of dextroamphetamine in humans. Psychopharmacology.

[106] Maeda T., Kiguchi N., Fukazawa Y., Yamamoto A., Ozaki M., Kishioka S. (2007). Peroxisome proliferator-activated receptor gamma activation relieves expression of behavioral sensitization to methamphetamine in mice. Neuropsychopharmacology.

[107] Schmitz J.M., Green C.E., Hasan K.M., Vincent J., Suchting R., Weaver M.F., Moeller F.G., Narayana P.A., Cunningham K.A., Dineley K.T. (2017). PPAR-gamma agonist pioglitazone modifies craving intensity and brain white matter integrity in patients with primary cocaine use disorder: A double-blind randomized controlled pilot trial. Addiction.

[108] Jones J.D., Comer S.D., Metz V.E., Manubay J.M., Mogali S., Ciccocioppo R., Martinez S., Mumtaz M., Bisaga A. (2017). Pioglitazone, a PPARgamma agonist, reduces nicotine craving in humans, with marginal effects on abuse potential. Pharmacol. Biochem. Behav..

[109] Nair M., Jayant R.D., Kaushik A., Sagar V. (2016). Getting into the brain: Potential of nanotechnology in the management of NeuroAIDS. Adv. Drug Deliv. Rev..

[110] Nair K.G., Ramaiya V., Sukumaran S.K. (2018). Enhancement of drug permeability across blood brain barrier using nanoparticles in meningitis. Inflammopharmacology.

[111] Huang Q., Chen Y., Zhang W., Xia X., Li H., Qin M., Gao H. (2024). Nanotechnology for enhanced nose-to-brain drug delivery in treating neurological diseases. J. Control. Release.

[112] Huang Q., Chen X., Yu S., Gong G., Shu H. (2024). Research progress in brain-targeted nasal drug delivery. Front. Aging Neurosci..

[113] Fu Q., Liu Y., Peng C., Muluh T.A., Anayyat U., Liang L. (2024). Recent advancement in inhaled nano-drug delivery for pulmonary, nasal, and nose-to-brain diseases. Curr. Drug. Deliv..

[114] Yin X., Zhang S., Lee J.H., Dong H., Mourgkos G., Terwilliger G., Kraus A., Geraldo L.H., Poulet M., Fischer S. (2024). Compartmentalized ocular lymphatic system mediates eye-brain immunity. Nature.

